# Opportunities and Challenges in Catheter-Based Irreversible Electroporation for Ventricular Tachycardia

**DOI:** 10.3390/pathophysiology31010003

**Published:** 2024-01-10

**Authors:** Matthew Leonard Repp, Ikeotunye Royal Chinyere

**Affiliations:** 1 College of Medicine, University of Arizona, Tucson, AZ 85724, USA; matthewrepp@arizona.edu; 2 Department of Medecine, Banner University Medicine, Tucson, AZ 85724, USA; 3 Sarver Heart Center, University of Arizona, 1501 North Campbell Avenue, Room 6154, Tucson, AZ 85724, USA

**Keywords:** cardiac, arrhythmia, ablation, catheter, ventricle, pulsed-field ablation

## Abstract

The use of catheter-based irreversible electroporation in clinical cardiac laboratories, termed pulsed-field ablation (PFA), is gaining international momentum among cardiac electrophysiology proceduralists for the non-thermal management of both atrial and ventricular tachyrhythmogenic substrates. One area of potential application for PFA is in the mitigation of ventricular tachycardia (VT) risk in the setting of ischemia-mediated myocardial fibrosis, as evidenced by recently published clinical case reports. The efficacy of tissue electroporation has been documented in other branches of science and medicine; however, ventricular PFA’s potential advantages and pitfalls are less understood. This comprehensive review will briefly summarize the pathophysiological mechanisms underlying VT and then summarize the pre-clinical and adult clinical data published to date on PFA’s effectiveness in treating monomorphic VT. These data will be contrasted with the effectiveness ascribed to thermal cardiac ablation modalities to treat VT, namely radiofrequency energy and liquid nitrogen-based cryoablation.

## 1. Introduction

**Figure 1:**
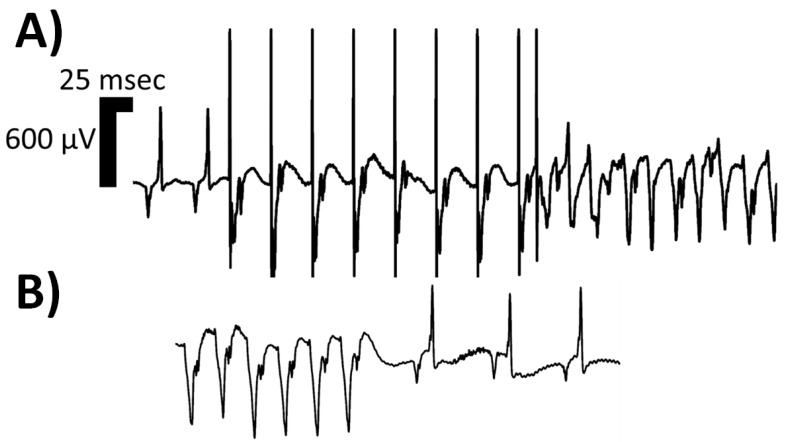
Isolated limb lead of a single rodent’s surface electrocardiogram depicting one morphologic classification of sustained ventricular tachycardia (VT), namely monomorphic VT. In Panel (**A**), two sinus complexes begin the rhythm strip, followed by an eight (8) S1–S2 programmed electrical stimulation (PES) drivetrain protocol, executed at twice diastolic-threshold on the epicardial surface using microelectrodes; the resulting monomorphic VT can be appreciated with P-waves continuing at the intrinsic sinoatrial node rate. In Panel (**B**), the spontaneous termination of the monomorphic VT is depicted, with a sinus pause and resumption of intrinsic conduction system activity at a slower rate than pre-PES. The rat suffered from heart failure with reduced ejection fraction induced via permanent left coronary artery ligation. With the onset of the induced monomorphic VT, the rodent’s invasive blood pressure readings no longer exhibited pulsatility, and the mean arterial pressure dropped. Abbreviations: msec—milliseconds; μV—microvolts.

**Figure 2:**
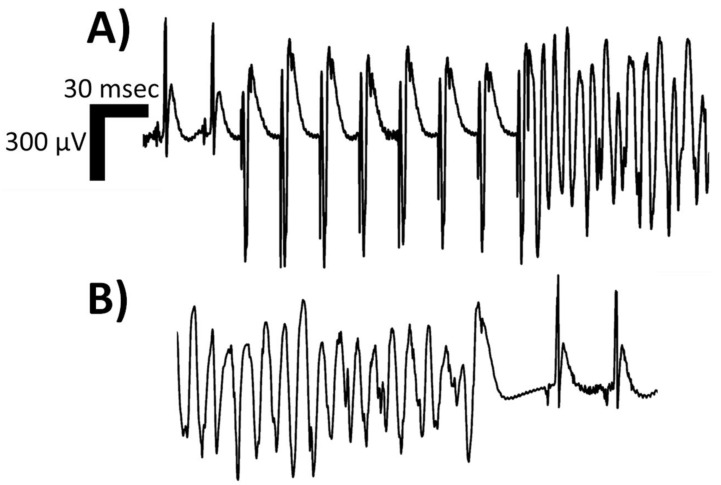
Isolated limb lead of a single rodent’s surface electrocardiogram depicting one morphologic classification of sustained ventricular tachycardia (VT), namely polymorphic VT. In Panel (**A**), two sinus complexes begin the rhythm strip, followed by an eight (8) S1–S2 programmed electrical stimulation (PES) drivetrain protocol, executed at twice diastolic-threshold on the epicardial surface using microelectrodes; the resulting polymorphic VT can be appreciated with P-waves continuing at the intrinsic sinoatrial node rate. In Panel (**B**), the spontaneous termination of the polymorphic VT is depicted, with a sinus pause and resumption of intrinsic conduction system activity at a slower rate than pre-PES. The rat suffered from heart failure with reduced ejection fraction induced via permanent left coronary artery ligation. With the onset of the induced polymorphic VT, the rodent’s invasive blood pressure readings no longer exhibited pulsatility, and the mean arterial pressure dropped. Abbreviations: msec—milliseconds; μV—microvolts.

Ventricular tachycardia (VT) is a wide-complex tachyrhythm (QRS duration on surface electrocardiogram > 120 milliseconds) that disrupts normal sinus rhythm and may lead to hemodynamic instability. This arrhythmia originates from within the ventricles and can be clinically defined as ≥3 consecutive ventricular beats occurring at ≥100 beats per minute. VT is categorized by the duration of the episode and the QRS morphology. Based on duration, VT is divided into non-sustained VT and sustained VT. Non-sustained VT terminates spontaneously within 30 s, whereas sustained VT lasts longer than 30 s or requires termination due to hemodynamic instability in <30 s [1]. Based on QRS morphology, VT can be classified as monomorphic (mmVT) or polymorphic. On electrocardiographic assessment, mmVT consists of a singular, consistent QRS morphology with minimal beat-to-beat variation (Figure 1), while polymorphic VT depicts varying QRS morphologies beat-to-beat (Figure 2) [1,2]. The most common cause of mmVT is structural sequelae from ischemic heart disease, but it may also arise from non-ischemic cardiomyopathies, iatrogenic etiologies, Purkinje system defects, or idiopathic etiologies [3]. Several causes of polymorphic VT exist, including R-on-T phenomenon, acute myocardial ischemia, congenital short QT syndrome, acquired long QT syndrome, catecholaminergic, torsade de pointes, bidirectional VT [4], and Brugada syndrome.

Pulsed-field ablation (PFA) is a tunable non-thermal ablative technique that, with the appropriate settings, is capable of inducing irreversible cell death via phospholipid bilayer electroporation in selective cardiac tissue populations with minimal damage to anatomically adjacent structures. This technique has recently been adapted to catheter-based technology to allow for clinical use in cardiac electrophysiology laboratories, though its use for research applications such as transmembrane transportation of relatively large macromolecules (ex.: plasmids) dates back to the early 1980s [5]. Furthermore, its use for clinical applications, such as selectively destroying malignant cells in oncology patients, dates back to the early 1990s [6]. PFA has demonstrated in pre-clinical [7] and non-randomized clinical [8] studies an acceptable degree of efficacy with respect to preventing atrial fibrillation recurrence over a short interval (approximately 1 year) while also maintaining an exceptionally favorable safety profile through the avoidance of collateral damage typically observed with post-thermal catheter ablation techniques.

Recently published case reports have illuminated the potential for PFA utilization for substrate suppression in the setting of recurrent ventricular tachycardia secondary to multiple underlying etiologies [9,10,11,12,13]. These preliminary reports likely signal the beginning of an expansion of the clinical electrophysiologists’ armamentarium to include a non-thermal catheter-based therapy in addition to the well-described catheter-based radiofrequency, cryoballoon, and laser options. As such, a careful review of the literature published to date is warranted in order to expedite regulatory approval in the United States of America [14], maximize the proportion of clinical decisions backed by evidence, and support the eventual generation of evidence- and consensus-based clinical guidelines for the use of PFA for ventricular tachyrhythms. In the present review, we aim to comprehensively review the pathophysiological mechanisms underlying VT, summarize the pre-clinical and adult clinical data published to date on PFA’s effectiveness in treating mmVT, and contrast these data with the effectiveness ascribed to thermal cardiac ablation modalities in the treatment of mmVT.

## 2. Pathophysiology of Ventricular Tachycardia

### 2.1. Etiologies Leading to Ventricular Tachycardia

#### 2.1.1. Myocardial Infarction, Adverse Remodeling, and Re-Entry

Sustained mmVT is nearly exclusively found secondary to the adverse ventricular remodeling associated with acute- or chronic ischemic heart disease. Adverse ventricular remodeling is frequently observed months to years post-acute myocardial infarction without appropriate pharmacologic suppression, where previously healthy contractile myocytes become irreparably damaged and are eventually replaced by myofibroblast-mediated fibrous tissue [15,16]. Within this myocardial scar are bundles of stunned cardiomyocytes with poor intercellular coupling and subsequently exhibit delayed electrical conduction.

**Figure 3:**
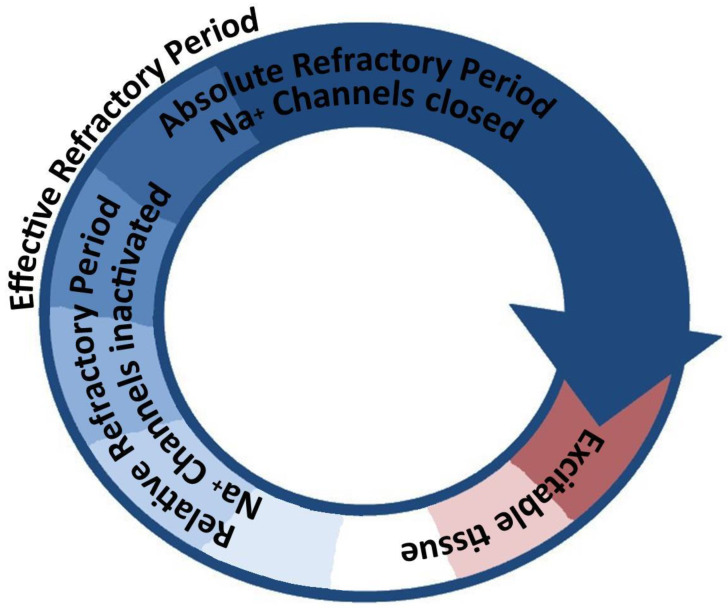
A stylized diagram illustrating the concept of arrhythmogenic re-entry. A circuit (not necessarily circular in shape, illustrated as such for simplicity) that contains a continuous path of conductive tissue, spatially separated in any of the three physical dimensions, that exhibits a heterogenous distribution of conduction velocities and/or repolarization rates can facilitate re-entry. A sufficiently large circuit, enabled either by anatomic (fixed) tissue separation or by functional (slowed conduction velocity) tissue separation, can allow a unified depolarization wavefront (arrowhead) to continuously meet excitable tissue and thus the circuit to activate with a certain cycle frequency. Abbreviations: Na^+^—sodium.

The presence of non-conductive tissue with spatially distributed pockets of conductive myocardium that have impaired repolarization creates a substrate for re-entry [17,18,19]. The criteria for anatomic re-entry are satisfied, namely, a fixed anatomic obstacle mediated by the focus of scar tissue, a circuit-like excitation wavefront pathway through impaired bundles, and unidirectional conduction block facilitated by locally prolonged repolarization in the setting of globally heterogenous repolarization [20] (Figure 3). A myocardial scar provides a fixed arrhythmogenic substrate and a single ventricular focus that consequentially favors mmVT pathophysiology [3].

In the acute phase of myocardial ischemia, transient sub-clinical ischemia and/or therapeutic reperfusion in acute coronary syndrome (ACS) can cause regional variations in myocyte membrane voltage stability. This instability can lead to ectopic depolarizations or increased automaticity described as R-on-T, Ashman phenomenon, or long-short coupling. These irregular, unregulated focal depolarizations can act as a nidus for triggered activity and initiate hemodynamically significant ventricular arrhythmias [21]. Although VT from ACS is predominately polymorphic, early studies have described an increased risk of mmVT with superimposed acute ischemia on a healed myocardial scar [22,23].

#### 2.1.2. Congenital and Acquired Cardiomyopathies

The incidence of mmVT is certainly higher in ischemic cardiomyopathy relative to non-ischemic; however, cases have been reported [24]. Akin to scar formation from myocardial infarction, inflammatory and degenerative processes can also lead to fibrotic tissue replacement of previously healthy myocytes, thus predisposing to the re-entrant form of VT. Causes of non-ischemic cardiomyopathy are vast and include familial cardiomyopathies such as arrhythmogenic right ventricular cardiomyopathy and non-compaction, autoimmune conditions such as cardiac sarcoidosis and cardiac amyloidosis, or infectious etiologies such as untreated Chagas disease and chronic viral myocarditis. Patients with established non-ischemic cardiomyopathies exhibit a surprisingly high incidence of mmVT via scar-related re-entry mechanisms, as evidenced by the seminal study from Marchlinski et al. [25].

#### 2.1.3. Iatrogenic Causes

Congenital heart disease (CHD) that is incompatible with life and consequently requires surgical repair increases the risk for life-threatening arrhythmias. The majority of VT in CHD occurs predominantly by re-entry mechanisms and is quintessentially illustrated in early-to-middle-aged adults with repaired tetralogy of Fallot (rTOF). Well-defined anatomic isthmuses bordered by regions of unexcitable tissue are created by the numerous suture lines created by the congenital heart surgeons to the anatomical barriers and slowed conduction necessary for re-entry circuits [26]. The arrhythmogenic substrates in rTOF include postsurgical scars following right ventricular outflow tract incisions, valve annuli, and patches [27]. In addition to the expected post-operative surgical scarring, patients with CHD can also develop VT due to adverse ventricular remodeling from impaired function, increased workload, or subsequent conduction system destruction.

#### 2.1.4. Purkinje System Disease

Bundle-branch re-entrant VT (BBR-VT) accounts for approximately 8% of sustained mmVTs [28] and can involve the right/left bundle, their branches, or the His bundle. Bundle-branch diseases, notably left-bundle branch blocks (LBBBs), facilitate interventricular conduction delays, which lead to ventricular contraction desynchrony and, if sufficiently severe, chronic, or hemodynamically significant, can lead to myocardial fibrosis via activation of the renin–angiotensin–aldosterone axis due to impaired perfusion of the periphery. The diffuse scar tissue deposition from this systemic process can provide an arrhythmogenic substrate for the formation of re-entrant circuits. These abnormalities disrupt the intrinsic cardiac conduction system, further increasing the likelihood of ventricular arrhythmias, including mmVT.

#### 2.1.5. Idiopathic Ventricular Tachycardia

Idiopathic VT is a small subset of tachyarrhythmias that occurs in patients without structural heart disease. The VT focus can be located anywhere in the heart but predominantly arises from the right ventricular outflow tract (RVOT) and, less commonly, from the left ventricular outflow tract [29]. RVOT-VT is frequently precipitated by high adrenergic states such as exercise, intense emotions, and illness [30]. Individuals affected by idiopathic VT are typically female, young, and healthy and thus require a thorough diagnostic workup for other causes and then for underlying heart disease. Unlike other causes of VT, this type is relatively benign given its transient nature in otherwise unremarkable cardiac systems and thus is associated with a low risk of sudden cardiac death [31].

### 2.2. Molecular Mechanisms of Monomorphic Ventricular Re-Entry

#### 2.2.1. Calcium Handling

Under optimal cardiomyocyte conditions, a sodium influx-mediated membrane action potential initiates the opening of voltage-gated L-type Ca^2+^ channels (LTCCs), leading to Ca^2+^ release from the sarcoplasmic reticulum (SR), facilitating allosteric manipulation of thin filament regulatory proteins and actin–myosin cross-bridge cycling. LTCCs play a vital role in maintaining membrane depolarization throughout the plateau phase of the action potential. For this reason, the L-type calcium current (*I*_Ca,L_) is crucial for preserving optimal action potential duration (APD) and illustrates why any alternations in *I*_Ca,L_ kinetics carry a high arrhythmogenic potential.

#### 2.2.2. Action Potential Prolongation and Repolarization Heterogeneity

In structural heart disease, a myriad of compensatory and pathophysiologic electrophysiological alterations ensue, precipitating a persistent proarrhythmic state. Ischemia-mediated calcium dysregulation (via sarco-endoplasmic reticulum adenosine triphosphate-ase expression downregulation and allosteric inhibition in addition to uncontrolled calcium sparks from failing ryanodine receptor clusters) and impaired potassium efflux [18] can directly lead to apoptosis via caspase activation or can lead to an impaired resting membrane potential and subsequent cell death. These two electrolyte abnormalities increase APD and ultimately exacerbate the repolarization reserve to depletion. Under conditions of acute metabolic stress, this low reserve state can lead to electrical alternans and, subsequently, mechanical alternans [32] as well as increase the likelihood of re-entrant arrhythmia via furthering the global cardiac repolarization heterogeneity.

This dispersion of repolarization can allow early afterdepolarizations, occurring during phases two or three of the cardiac action potential near the absolute refractory period, or the higher risk delayed afterdepolarizations occurring during phase four in the relative refractory period, to perturb the systems sufficiently to activate a dormant re-entrant system.

#### 2.2.3. Dysregulated Na^+^ Handling

The rapid upstroke of phase one in the cardiomyocyte action potential is primarily attributed to the influx of sodium ions (Na^+^) through the sodium current (*I*_Na_), making it predominantly responsible for tissue conduction velocity. In myocytes battered by local hypoxia and impaired membrane voltage regulation, the properties of the *I*_Na_ fail to completely inactivate and/or close throughout the action potential, resulting in late *I*_Na_. This late depolarizing current has been demonstrated to be induced by the Ca^2+^/calmodulin-dependent protein kinase II pathways, which are activated in the presence of structural heart disease to facilitate salvaging cardiac function via calcium desensitization [33]. The heightened intracellular Na^+^ concentration subsequently triggers an additional increase in cytosolic Ca^2+^ levels via membrane-bound Na^+^-Ca^2+^ exchangers. These mechanisms collectively contribute to APD dispersion and elevated arrhythmogenic risk [18].

## 3. Pulsed-Field Ablation for Arrhythmogenic Substrate Suppression

### 3.1. Regulatory Status of Clinical Pulsed-Field Ablation

PFA achieved regulatory approval for clinical use in the European Medicines Agency when Farapulse Inc., a subsidiary of Boston Scientific, achieved “Conformité Européene” in January of 2021 for the treatment of paroxysmal atrial fibrillation in adults [34] and shortly thereafter in March of 2021, Medtronic attained the same approval for their Affera™ Mapping and Ablation System. Though the same systems are also being utilized in the United States of America for various clinical conditions, including persistent left superior vena cava [35], neither has yet to achieve regulatory approval for clinical use by the Food and Drug Administration nor the Chinese National Medical Products Administration, though investigations are pending [36]. A third system, the Galaxy Medical/Galvanize Therapeutics CENTAURI™ System, attained European regulatory approval in August of 2022 [37], making it the most recent approval to date. As many as thirteen additional PFA systems are still in the development pipeline; however, no information regarding regulatory pursuits for pediatric indications can be found, though clinical case reports are arising [38].

### 3.2. Foundational Findings Supporting Pulsed-Field Ablation

#### 3.2.1. Mechanism of Action

Irreversible electroporation is the process of exposing a cell’s phospholipid bilayer membrane to nanosecond strong “non-thermal” [39] electric fields (voltage) to overwhelm the membrane’s inherent electrical capacitance threshold and creating sufficiently large membrane pores that cell death via necrosis or apoptosis. This process is tunable in that the voltage, pulse duration, pulse frequency, drivetrain, and application site can be adjusted independently; the optimization of these settings of cardiac catheter-based ablation is referred to as pulsed-field ablation. Electroporation requires cell membranes and thus creates specificity for biologically active membranes within the relatively precise electric field, thus sparing non-membranous connective tissue and support matrices. In addition, the small field of action minimizes damage to biologically active tissues outside of the electric field, such as nervous tissue, though variable non-fatal damage and regeneration have been described [40]. In addition, the non-thermal mechanism of action does not denature tissue or activate any damage-sensitive innate receptors for non-specific inflammation [41]. A minimized inflammatory response yields less fibrosis and off-target tissue damage. This non-thermal technique was adapted to force-sensing catheter-based systems and ultimately made uniform with a “single shot” operation, which avoids any theoretical cancellation effects from rapid serial dosing [42].

#### 3.2.2. Preclinical Proof-of-Concept

Published data evaluating the molecular effects of PFA on cell cultures have revealed a differential response and toxicity threshold from both murine and human cardiomyocytes, neurons, and cardiac adipocytes based on electric field strengths [43,44,45,46,47]. Human esophageal smooth muscle cells have also been assessed and exhibited a greater resistance to FPA [48]. Dose-response assessments have been generated for isolated rodent ventricles [49]. In addition to differences in population thresholds, in silico models are optimized to maximize myocytes’ toxicity based on cell orientation relative to the electric field [50] and tissue fiber orientations [51].

Langendorff and whole-animal studies have been completed in swine [52,53,54,55,56,57], canine [58], ovine [59], and even rabbit [60] models using various PFA catheter types and methodologies. Consensus findings include the creation of transmural lesions via concentrated apoptosis with no evidence of thermal-based tissue damage. Though most articles do not document any adverse effects from PFA, coronary artery spasms and chronic stenosis via neointimal hyperplasia have been appreciated.

### 3.3. Pulsed-Field Ablation for Monomorphic Ventricular Tachycardia

#### 3.3.1. Pre-Clinical Efficacy for Monomorphic Ventricular Tachycardia

Unfortunately, no pre-clinical data assessing the safety, efficacy, or reproducibility of PFA for VT exist in animal models of human cardiac disease. However, pre-clinical reports on the efficacy of ventricular PFA [53,56,57,61], as well as the safety of ventricular PFA [62], are increasing. All five studies were conducted in swine [53,56,57,61,62] and had short-term endpoints (ranging from 2 to 7 days [57] to 6 to 8 weeks [53]). Sample sizes were small (ranging from 4 to 10 swine), though appropriate for preliminary de novo safety study designs. Translational studies in models that closely recapitulate the pathophysiological processes encountered in the clinical cardiac electrophysiology lab (namely rodent [17] and swine models of ischemic cardiomyopathy assessed with clinically relevant programmed electrical stimulation techniques) are needed to carefully detail the immediate-, short-, and long-term efficacy of PFA for mmVT.

#### 3.3.2. Adult Clinical Efficacy for Monomorphic Ventricular Tachycardia

Clinical data for the management of patients suffering from VT are presently limited to the case report and case series stage of development [9,10,11,12,13]. Though the etiologies varied from scar-mediated [9,10,11,13] to ventricular aneurysm [11], and arrhythmogenic cardiomyopathy [11], atrioventricular malformations [12], non-ischemic cardiomyopathy [13,63], and even ectopy [11,64], no adverse events were reported in any of these observational studies (Table 1). Of note, the longest time period that a patient was monitored for potential arrhythmia recurrence post-PFA was 6 months [9]. Though concerns for coronary artery vasospasm and/or stenosis [65,66] secondary to neointimal hyperplasia, as well as nerve damage [67], have been raised, the volume-adjusted incidence suggests a lower risk of these neurovascular complications compared to the thermal catheter-based ablation modalities used for comparable substrates.

#### 3.3.3. Outcome Comparison with Thermal Cardiac Ablation

Due to the preliminary stage of PFA for mmVT management and likely also due to the publication bias for positive-result studies, all (6/6, 100%) case reports and case studies published to date describe success with regard to the primary outcome of arrhythmia cessation and/or failed VT induction. The success rate for managing mmVT secondary to coronary disease over a six-month time interval has been approximated to 62% [68]. Laser energy continues to be investigated at the pre-clinical level for ventricular rhythm applications [69]; nonetheless, its use remains focused on lead extractions due to operator familiarity with radiofrequency energy. Similarly, cryoballoons have been utilized for ventricular substrates at the case series level [70] but are rarely used for monomorphic ventricular tachycardia, and thus, no success rates nor complication rates can be confidently assessed.

## 4. Conclusions

Chronic compensatory changes post-ischemic insult produce ischemia-mediated calcium dysregulation, impaired potassium efflux, global action potential duration dispersion, and increased arrhythmogenic risk. These electrophysiologic changes paired with structural scar tissue can propagate the initiation of ventricular tachyrhthms, including monomorphic ventricular tachycardia (mmVT). Though current management of pharmacologically resistant mmVT includes catheter ablation with a thermal mechanism of action, recurrence occurs, and anatomical challenges and safety risks must be carefully considered. Irreversible electroporation provides a non-thermal option for the invasive cardiac electrophysiologist and may increase the overall efficacy of catheter ablation for difficult-to-treat or anatomically limiting cardiac tachyrhythms. At the present time, pre-clinical data supporting pulsed-field ablation (PFA) for mmVT are lackluster; however, clinical case reports are mounting. Additional work regarding the safety, efficacy, and long-term durability of PFA for mmVT is needed.

## Figures and Tables

**Table 1:** A table highlighting the pertinent qualities of the clinical case reports on the use of pulsed-field ablation for ventricular tachycardia. Case reports/series are listed in order of reference number and include the PFA technology parameters, if available. Abbreviations: Ref—reference, M—male, F—female, PFA—pulsed-field ablation, MI—myocardial infarction, ICD—implantable cardioverter defibrillator, defibs—defibrillations, VT—ventricular tachycardia, mmVT—monomorphic ventricular tachycardia, ATP—anti-tachycardia pacing, mm—millimeters, kV—kiloVolts, HFrEF—heart failure with reduced ejection fraction, LVEF—left ventricular ejection fraction, RFA—radiofrequency ablation, CRT-D—cardiac resynchronization therapy-defibrillator, aRV—atrialized right ventricle, RV—right ventricle, LAD—left anterior descending coronary artery, W—watts, C—Celsius, RVOT—right ventricular outflow tract, PVCs—premature ventricular contractions, sec—seconds, LV—left ventricle.

Research Group [Ref. #]	Patient Age, Gender	Cardiovascular Comorbidities	Arrhythmogenic Nidus	Prior Procedures	PFA Indication	PFA Catheter Type	PFA Parameters
Ouss et al. [9]	69, M	Remote Anterior MI	Distal anteroseptum	2 RFA attempts; ICD	Recurrent VT requiring ATP	Farawave™ Pentaspline (31 mm)	2.0 kV via biphasic waveform (7 overlapping applications)
Martin et al. [10]	68, M	HFrEF (LVEF 30%) Ischemic cardiomyopathy	Posterior, sub-aortic valve intramural circuit	Peri-aortic endocardial and epicardial RFA attempts; CRT-D	Treatment-resistant mmVT requiring ATP	Farawave™ Pentaspline (31 mm)	2.0 kV via 5 biphasic/bipolar pulses (56 applications)
Lozano-Granero et al. [11]	83, M	Non-obstructive coronary artery disease	Mid-apical lateral LV wall	None	Sustained mmVT	Farawave™ Pentaspline (31 mm)	2.0 kV via 4 biphasic/bipolar microsecond pulses (9 applications)
	83, F	LV aneurysm	LV aneurysm	2 RFA attempts; ICD	Treatment-resistant electrical storm requiring ATP and defibs	Farawave™ Pentaspline (31 mm)	2.0 kV bipolar pulses (18 applications)
	69, M	Arrhythmogenic cardiomyopathy with biventricular involvement	Basal inferolateral free LV wall	ICD	Treatment-resistant RV VT requiring defibs	Farawave™ Pentaspline (31 mm) + RFA: Thermocool Smarttouch^®^	2.0 kV bipolar pulses (12 applications) + RFA: Single 40.0 W application
Krause et al. [12]	33, M	Ebstein’s anomaly	Anterior junction between aRV and RV	1 RFA attempt; ICD	Recurrent mmVT requiring defibs	Farawave™ Pentaspline (31 mm)	Parameters not specified (35 applications)
Adragão et al. [13]	60, M	Remote LAD MI HFrEF (LVEF <20%)	Extensive anterior wall/septum scar	3 endocardial RFA attempts; CRT-D	Treatment-resistant electrical storm with multiple VTs	Farawave™ Pentaspline (31 mm) + QDOT Micro™	2.0 kV at 6 septal sites (18 applications) + RFA: 50 W; 50 °C; Index 600
Weyand et al. [63]	61, M	Non-ischemic dilated cardiomyopathy	LV basal anterolateral endocardial scarring	1 endocardial RFA attempt; ICD	Electrical storm with mmVT	Thermocool Smarttouch^®^	Biphasic/unipolar (“Several” applications)
Schmidt et al. [64]	48, F	No structural heart disease	RVOT	None	Medication-refractory symptomatic PVCs	Farawave™ Pentaspline (31 mm)	2.5 sec pulses at 1.8 kV (8 applications)

## Data Availability

All data utilized in this review article are publicly available.
